# 肺癌血清肿瘤标志物的临床意义

**DOI:** 10.3779/j.issn.1009-3419.2011.03.16

**Published:** 2011-03-20

**Authors:** 肖 赵, 孟昭 王

**Affiliations:** 100730 北京，中国医学科学院，中国协和医科大学，北京协和医院呼吸内科 Department of Respiratory Medicine, Peking Union Medical College Hospital, Chinese Academy of Medical Sciences and Peking Union Medical College, Beijing 100730, China

**Keywords:** 肺肿瘤, 肿瘤标志物, 诊断, 治疗, Lung neoplasms, Biological tumor marker, Diagnosis, Treatment

## Abstract

肺癌血清肿瘤标志物在肺癌的早期诊断、病理分型、疾病分期、疗效评估和指导预后等方面起着重要的作用，本文旨在对神经元特异性烯醇化酶（NSE）、胃泌素释放肽（ProGRP）、细胞角蛋白19片段（CYFRA 21-1）、组织多肽抗原（TPA）、鳞状细胞癌相关抗原（SCC-Ag）和癌胚抗原（CEA）这6种重要的肺癌血清肿瘤标志物的临床意义进行综述。

## Abstract (en)

Lung cancer shows a tendency of higher incidence and higher mortality in recent years, but the overall 5-year survival rate is less than 15%. Serum tumor markers of lung cancer play an important role in early diagnosis, determining of pathology types, staging, evaluation of response, and prognosis of lung cancer. In this review, 6 most important markers were reviewed, including neuron-specific enolase (NSE), pro-gastrin-releasing peptide (ProGRP), cytokeratin 19 fragments (Cyfra 21-1), tissue polypeptide antigen (TPA), squamous cell carcinoma associated antigen (SCC-Ag), carcinoembryonic antigen (CEA).

进入21世纪以后，肺癌已经超过胃癌和肝癌成为我国恶性肿瘤死因的第一位^[1]^。非小细胞肺癌（non-small cell lung cancer, NSCLC）占肺癌的85%，5年生存率仅15%。小细胞肺癌（small cell lung cancer, SCLC）的2年生存率仅为1%。70%的肺癌患者在确诊时已经是局部晚期或远处转移（Ⅲb/Ⅳ期），失去了手术机会，缺乏有效的治疗手段^[2]^。如果肺癌患者能在Ia期被诊断，那么5年生存率可提高到80%^[3]^。因此肺癌的早期诊断成为我国临床医生和科研工作者的重要任务。

肿瘤标志物（tumor markers, TMs）是一类在肿瘤发生、发展过程中，由肿瘤细胞所产生和分泌并释放到血液、体液、组织中反映肿瘤存在和生长的一类物质。好的肿瘤标志物应具有较高的灵敏度和特异性，不但有助于癌症的早期诊断、病理类型的判断和癌症分期，更重要的是能够评估治疗效果和预后并指导个体化治疗。本文对目前应用于临床的6种血清肿瘤标志物进行综述，包括神经元特异性烯醇化酶（neuron-specific enolase, NSE）、胃泌素释放肽前体（pro-gastrin-releasing peptide, ProGRP）、细胞角蛋白19片段（CYFRA 21-1）、组织多肽抗原（Tissue Polypeptide Antigen, TPA）、鳞状细胞癌相关抗原（squamous cell carcinoma related antigen, SCC-Ag）和癌胚抗原（carcinoembryonic antign, CEA）。

## 1 神经元特异性烯醇化酶（NSE）

NSE是神经源性细胞分泌的一种蛋白酶，在神经内分泌肿瘤中明显升高。正常人和良性疾病患者的平均NSE血清浓度为（4.2±1.1）ng/mL。一般 > 12.3 ng/mL-13 ng/mL则认为升高。NSE在SCLC增高明显，在NSCLC也可升高。一些良性疾病和其它器官恶性肿瘤中NSE也可升高，如感染性疾病和甲状腺髓样癌、黑色素瘤、神经母细胞瘤、嗜铬细胞瘤、胰岛细胞瘤、视网膜母细胞瘤等。

一些研究^[4-7]^认为NSE对SCLC的诊断敏感性达64%-74.5%，主要对广泛期患者有较好的诊断作用，但这些研究样本量较小。Shibayama等^[8]^比较了114例SCLC、142例NSCLC、103例肺良性疾病患者和108例正常人的血清NSE浓度，NSE对SCLC的诊断敏感性仅有43%，低于之前的报道。作者认为主要是由于该研究中局限期SCLC占60.5%，而NSE对局限期SCLC的诊断敏感性仅有20.3%。

血清NSE浓度不仅对SCLC的诊断有提示作用，对其治疗效果和预后也有预测作用。Shibayama等^[8]^研究比较了27例血清NSE水平升高的SCLC患者和47例水平正常的SCLC患者在接受化疗后的客观完全缓解率（18.5% *vs* 61.7%, *P* < 0.001）、中位生存期（10.5个月*vs* 21.3个月, *P*=0.003）和5年生存率（3.7% *vs* 27.5%, *P*=0.003），结果显示了NSE的升高提示预后不良和较短的生存期。

NSE对SCLC的复发也有一定的预测价值。Niho等^[9]^研究显示，在66例复发的SCLC患者中，53%的患者血清NSE水平升高，但NSE并不是复发的强预测因子。在Okusaka等^[10]^的研究中，44例SCLC中26例复发，其中19例NSE水平升高，并且19例中有7例（39%）接受一线化疗后NSE血清水平下降，复发时又重新升高（NSE升高定义为连续两天NSE升高水平较前次测定增加 > 10%，或一次测定较前增加 > 50%）。这7例患者血清NSE升高的时间较临床复发平均晚20天（-85天-124天）。血清NSE的水平在治疗后是否变化对生存期并无影响，多项研究^[9-11]^都得出了相同的结论，但样本量均较小，结论是否能扩展到所有SCLC患者需要较大样本量的研究。

NSE对NSCLC诊断的敏感性较低，仅有10%-20%的NSCLC患者血清NSE浓度升高（一般比正常值高1-2倍）。Molina等^[12]^测定了211例NSCLC患者的NSE水平，其中45（21.3%）例升高，无统计学差异；但多变量分析发现NSE的升高与NSCLC患者的生存期呈现负相关，可作为NSCLC患者生存期的独立预测因子。

Paone等^[13]^对50例NSCLC和17例SCLC的研究显示，NSE与CYFRA 21-1联用对鉴别SCLC和NSCLC有97%的准确率，特别是对进展期肺癌病理类型的判断有很大帮助。

## 2 胃泌素释放肽前体（ProGRP）

ProGRP是由胃肠道分泌的一种促胃泌素释放肽（GRP）前体，可存在于胎儿肺的神经内分泌细胞内。ProGRP血清浓度 > 46 pg/mL-50 pg/mL认为升高，而正常人和良性疾病患者一般为（21.7±9.1）pg/mL。在SCLC患者中ProGRP血清浓度升高明显，可达到（1, 673.9±706）pg/mL。如果ProGRP血清浓度 > 200 pg/mL，则应高度怀疑SCLC。研究^[14]^显示ProGRP血清中位浓度局限期SCLC患者为472 pg/mL，而广泛期SCLC患者高达1, 136 pg/ mL。ProGRP在一些良性疾病和其它恶性疾病中也会升高，如肾脏功能衰竭患者的ProGRP血清浓度可高达310 pg/mL，但一般仍低于SCLC患者。

ProGRP对SCLC诊断的敏感性在47%-86%之间^[6, 7]^。与NSE相比，ProGRP对于SCLC诊断的敏感性更高，尤其对于局限期SCLC。研究^[8]^显示ProGRP和NSE对SCLC的诊断敏感性分别为64.9%和43.0%（*P* < 0.01），特别对于局限期SCLC ProGRP更显示出明显的优势（56.5% *vs* 20.3%, *P* < 0.01）。

ProGRP对SCLC患者治疗效果评估和复发监测也起到一定的作用^[15]^。在Okusaka等^[10]^的研究中，26例复发SCLC患者中18例患者一线化疗前ProGRP血清浓度升高，接受化疗后ProGRP血清浓度下降，其中17例（94%）患者在疾病复发时再次升高。ProGRP血清浓度的升高平均较临床复发早35天（-95天-151天）。

Hirose等^[16]^对178例SCLC患者进行了血清ProGRP和NSE水平的研究。化疗有效后复发患者123例，其中103例患者治疗前血清ProGRP和/或NSE水平升高，在一线化疗完成时和复发时再次测定血清ProGRP或NSE水平。在复发的患者中，血清ProGRP和NSE水平升高的患者分别占69.3%（70/101）和60.2%（56/93）（*P*=0.38）；两项都升高的占45.1%（41/91），其中至少一项升高的占81.3%（74/91）。复发时血清ProGRP水平升高的患者中，其中63例（84%）治疗前血清ProGRP水平高于正常，只有7例（16%）患者治疗前血清ProGRP水平正常。对于没有复发的患者，均未观察到血清ProGRP和NSE水平的升高。在该研究中只有4例患者血清ProGRP或NSE水平升高发生在临床复发前，3例在临床复发2个月前出现血清学肿瘤标志物异常，1例发生在10个月前。复发时血清ProGRP水平升高对生存期并无影响（7个月*vs* 9个月，*P*=0.17）。而NSE可作为SCLC患者复发后生存期的独立预测指标，复发时血清NSE水平升高的患者较正常水平的患者生存期明显缩短（6个月*vs* 14个月，*P* < 0.001）。

ProGRP对SCLC患者的预后也有一定的评估作用，但对生存期的预测没有NSE有优势。有研究^[8]^测定了73例接受化疗的SCLC患者血清ProGRP浓度，并比较了血清ProGRP浓度和生存期的关系。47例血清ProGRP浓度升高和27例血清ProGRP浓度正常的SCLC患者相比中位生存期（16.4个月*vs* 25.7个月，*P*=0.038）和5年生存率（10.6% *vs* 32.9%, *P*=0.038）都较低，危险比（Hazard Ratio, HR）为1.65（95%CI: 0.86-3.19, *P*=0.134）。

NSE与ProGRP联合应用可提高SCLC的诊断率，对SCLC患者化疗疗效和生存期预测有很好的评估作用^[9-11, 17]^。

10%-30%左右的NSCLC患者的ProGRP血清浓度也会升高，鳞癌患者比腺癌患者升高程度更明显，但一般升高程度 < 120 pg/mL^[18, 19]^。Nisman等^[20]^测定了88例NSCLC患者的血清ProGRP和NSE浓度，通过单变量分析发现血清ProGRP浓度与NSCLC患者生存率有关（*P*=0.03）。

## 3 细胞角蛋白19片段（CYFRA 21-1）

细胞角蛋白19片段是细胞角蛋白中间丝的亚单位，表达于上皮细胞和上皮细胞来源的恶性肿瘤细胞。血清CYFRA 21-1正常值一般 < 1.5 ng/mL，如 > 3.2 ng/mL-3.6 ng/ mL认为阳性。NSCLC患者的血清CYFRA 21-1升高较明显，部分SCLC患者的血清CYFRA 21-1浓度也会升高。而ProGRP和NSE正常的SCLC的CYFRA 21-1一般并不升高。一些肺良性疾病CYFRA 21-1血清浓度也有可能升高。

以往文献报道血清CYFRA 21-1诊断NSCLC的敏感性差异较大，范围为23%-70%。但对于鳞癌，一般均认为CYFRA 21-1有较高的敏感性。CYFRA 21-1与CEA联合应用诊断NSCLC敏感性达81%，然而即使CEA和CYFRA 21-1都升高对NSCLC的诊断也不具特异性。

Ardizzoni等^[21]^对107例晚期NSCLC患者化疗前和2个疗程后的血清CYFRA 21-1和CEA浓度进行了测定和分析，结果显示患者血清CYFRA 21-1和CEA下降与影像学客观疗效相关，如浓度下降 > 20%可认为化疗有效，并且血清CYFRA 21-1和CEA浓度是生存的强独立预测因子。其它研究^[22, 23]^也得出了相似的结论。

Ando等^[24]^对57例SCLC患者的研究显示，NSE与CYFRA 21-1联用可对SCLC患者的预后进行预测，两者都升高的患者较仅NSE升高的患者死亡风险比明显升高。

联合ProGRP、NSE和CYFRA 21-1对于鉴别SCLC和NSCLC有重要意义。Lou等^[25]^测定了52例晚期转移肺癌患者的ProGRP、NSE和CYFRA21-1血清水平。SCLC患者ProGRP（*P* < 0.001）和NSE（*P* < 0.03）血清浓度明显升高。多变量分析显示联合这3种肿瘤标志物对于鉴别SCLC和NSCLC有很高的准确度（C Index=0.98）。

Pujol等^[26]^对128例NSCLC患者的研究显示鳞癌患者血清CYFRA 21-1浓度升高较明显，平均血清浓度达5.6 ng/mL，其它病理类型的肺癌患者血清CYFRA 21-1浓度仅轻度升高（平均血清浓度 < 4 ng/mL）；血清CY-FRA 21-1浓度与疾病严重程度有关，Ⅳ期NSCLC患者的CYFRA 21-1血清浓度升高明显，平均可达7.4 ng/mL，而局限期NSCLC患者血清CYFRA 21-1平均浓度仅为3.8 ng/ mL。2, 063例NSCLC患者的*meta*分析^[27]^显示CYFRA 21-1血清浓度高的患者生存期少于正常浓度的患者，可作为一项独立预后因素。

## 4 组织多肽抗原（TPA）

TPA是细胞角质蛋白8、18和19的循环多肽复合物，血清TPA浓度与细胞增殖和肿瘤生长速度有关。TPA对于肺癌的诊断并不具有特异性，在很多其它类型的恶性肿瘤中TPA血清浓度都可升高。一些良性疾病也可引起TPA的升高，如感染性疾病、急性肝炎、妊娠和一些自身免疫性疾病。Buccheri等^[28]^测定了226例肺癌患者的血清TPA浓度，其中137例（61%）水平升高（> 100 U/L），平均血清浓度为204 U/L。TPA升高水平与组织类型无关，但与肿瘤分期有关，TNM分期越晚血清TPA浓度越高。

TPA的升高也预示着预后不良。Matsuguma等^[29]^对344例完全切除的Ⅰ期NSCLC患者进行了前瞻性研究，其中12 %的患者术前血清TPA浓度升高，与血清TPA浓度正常的患者相比其5年生存率下降（66% *vs* 81%, *P*=0.031, 1）。

## 5 鳞状细胞癌相关抗原（SCC-Ag）

SCC-Ag是一种细胞结构蛋白，在各组织来源的鳞状细胞癌其血清浓度升高，皮肤病和肺部感染时也会升高。正常人的血清SCC-Ag浓度一般 < 1.5 ng/mL。SCC-Ag对NSCLC诊断的敏感性为15%-55%，而对SCLC的诊断敏感性仅有15.5%。在排除肾衰竭的情况下，SCC-Ag升高高度提示NSCLC可能。

SCC-Ag对于肺鳞癌患者的诊断敏感性较高。Molina等^[19]^的研究中，472例NSCLC患者中101例（21.4%）血清SCC-Ag浓度升高，平均浓度为（0.8±0.4）ng/mL，而175例SCLC患者的血清SCC-Ag均正常，平均浓度为（0.5±0.1）ng/mL；在310例良性疾病患者中有6例血清SCC-Ag浓度升高。其中75例鳞癌患者SCC-Ag血清浓度升高明显，50例为Ⅰ期-Ⅲ期鳞癌患者，平均SCC-Ag血清浓度为1.35 ng/mL，25例为Ⅳ期鳞癌患者，平均血清SCC-Ag浓度为2.5 ng/mL；290例非鳞癌患者中血清SCC-Ag浓度升高的患者仅占6.5%，平均血清SCC-Ag浓度 < 0.7 ng/mL。

对200例肺鳞癌患者的研究^[30]^显示，SCC-Ag对肺鳞癌的诊断敏感性为0.72（95%CI: 0.65-0.78），其中76%的患者SCC-Ag≤2.8 ng/mL，中位生存期为12个月；24%的患者SCC-Ag > 2.8 ng/mL，中位生存期为8个月；多变量分析显示SCC-Ag的升高对生存期并无影响（RR=1, 95%CI: 0.71-1.52）。

对354例（鳞癌占23%）完全切除的Ⅰ期NSCLC患者的研究^[29]^认为术前血清SCC-Ag浓度与患者预后相关，术前12 %的患者血清SCC-Ag浓度升高，与血清SCC-Ag浓度正常的患者相比5年生存率下降（63% *vs* 82%, *P*=0.004, 5）。

## 6 癌胚抗原（CEA）

CEA是由胃肠道细胞分泌的一类糖蛋白，可引起机体的免疫反应，其升高主要见于消化系统肿瘤，肺癌患者血清CEA也可升高。CEA经肝脏代谢，因此肝脏的良性疾病也可导致CEA升高，尤其是胆管阻塞和胆汁淤积。早期研究显示健康的吸烟者较非吸烟者血清CEA浓度高。文献报道正常血清CEA值为2.5 ng/mL-6.9 ng/mL，大部分研究CEA阳性判断值为5 ng/mL。良性疾病导致的CEA升高通常 < 10 ng/mL。

血清CEA浓度升高有助于肺癌的诊断。在Molina^[19]^的研究中，472例NSCLC患者其中256例（54.2%）血清CEA浓度升高，平均浓度为5.8 ng/mL；175例SCLC患者中84例（48%）血清CEA浓度升高，平均浓度为4.8 ng/ mL；而310例良性疾病患者中仅16例患者血清CEA浓度升高，平均浓度 < 3 ng/mL。182例鳞癌患者平均血清CEA浓度 < 6 ng/mL；205例腺癌患者CEA血清浓度升高明显，Ⅰ-Ⅲ期肺腺癌患者平均血清浓度为6.4 ng/mL，Ⅳ期肺腺癌患者平均血清CEA浓度为19.5 ng/mL。

CEA单独升高对NSCLC并无诊断意义，因为在其它很多恶性肿瘤中CEA都会升高。CEA和CYFRA 21-1联合应用对NSCLC的预测较有意义。联合CEA、SCC-Ag和Cyfra21-1对NSCLC的诊断和评估具有很高的敏感性。

Matsuguma等^[29]^测定了355例可完全切除的Ⅰ期NSCLC患者的血清CEA、Cyfra21-1、SCC、CA19-9、CA125、TPA、NSE、SLX浓度。研究显示在众多的NSCLC肿瘤标志物中，对手术完全切除的Ⅰ期患者进行预后评估CEA是最好的预测指标。与术前血清CEA浓度正常的患者相比，CEA升高的患者5年生存率下降（52% *vs* 89%, *P* < 0.000, 1），因此认为对于术前CEA升高的完全切除的Ⅰ期NSCLC患者，应密切随访或积极进行术后辅助化疗。

研究^[31]^测定了1, 000例临床Ⅰ期的NSCLC患者术前和术后的血清CEA浓度。其中术前632例正常，368例升高。在368例血清CEA浓度升高的患者中，242例术后降到正常水平以下。术前CEA水平正常和CEA水平升高患者的5年生存率分别是75.2%和53.8%（*P* < 0.000, 1）。术前CEA血清浓度升高的患者术后CEA水平也与生存率相关。术后CEA水平降到正常值以下的患者与术后CEA仍然升高患者的5年生存率分别为62.6%和35.2%。因此术后CEA水平仍升高，预示着预后不良，应密切随访。在该研究中有13例患者术前血清CEA浓度 > 50 ng/mL，虽然都是Ⅰ期患者，9例复发时便为远处转移，但3例患者生存期长于5年。

对于不可手术切除的晚期NSCLC患者，治疗前CEA血清浓度的升高也预示着较短的生存期。Cedres等^[32]^对320例（鳞癌23.8%、腺癌39.7%）NSCLC患者血清CEA水平和无疾病进展期、生存期的关系进行了研究。其中275例为进展期NSCLC，血清CEA水平升高的患者与CEA水平正常患者相比，无疾病进展期（5.3个月*vs* 7.4个月，*P*=0.011）和生存期（10.0个月*vs* 14.0个月，*P*=0.085）都缩短。

Pollan等^[33]^对390例NSCLC患者的研究显示，CEA血清浓度的升高对鳞癌患者的生存期并无影响，但对于腺癌患者和其它类型NSCLC，CEA血清浓度的升高预示着预后不良。

## 7 联合应用几种肿瘤标志物对于NSCLC和SCLC的鉴别

Molina等^[19]^测定了647例肺癌患者（NSCLC 472例，SCLC 175例）的多种肿瘤标志物，包括NSE、ProGRP、CYFRA 21-1、SCC、CEA、CA125，发现在这几种肿瘤标志物中ProGRP和CYFRA 21-1分别对SCLC和NSCLC的诊断敏感性最高。联合上述几种肿瘤标志物鉴别SCLC和NSCLC可达77.4%的准确率。Molina为临床工作者提供了一个实用的病理类型鉴别步骤，见图 1。

**1:**
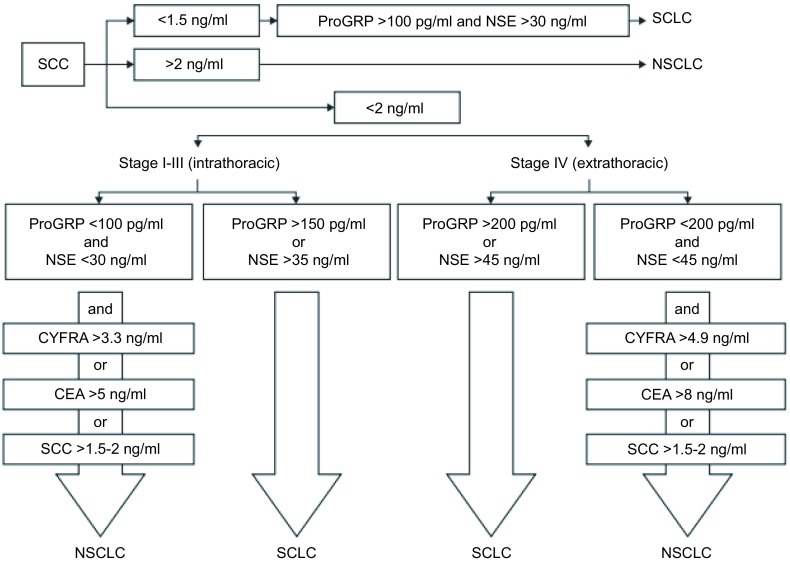
SCLC和NSCLC病理类型判断步骤 Algorithm to suggest the specific histological diagnosis using serum tumor markers

目前肿瘤标志物研究存还存在很多问题。肿瘤标志物在肿瘤的诊断、疗效评估和预后预测等方面主要作为辅助指标应用。近几十年寻找新的肿瘤标志物研究并无突破，正在研究中的肿瘤标志物很多，但是很少能应用于临床。目前肿瘤标志物研究中主要存在以下几个问题^[34, 35]^：临床试验均为回顾性的研究，没有事先设定明确的检验假设；试验的样本量较少，大多数临床试验结论没有统计学意义；临床试验中设定的主要观察指标并非最佳选择；由于研究方法、阳性判断值和主要观察指标的不同，各试验所得到的结论不具有可比性。

因此应在临床试验实施前完善试验设计，使试验的可信度更高，而且肿瘤标志物的研究应以应用于临床为目标。临床肿瘤研究试验评估方案（PACCT）中关于肿瘤标志物研究的指南草案^[36]^指出，临床研究设计应遵循以下步骤：确定潜在有意义的肿瘤标志物、临床试验设计的改进和细化、临床应用的初步评估、试验设计标准化、试验和临床应用的最终确认。

今后对于肿瘤标志物的研究应向以下方向发展：①通过大规模临床实验验证肿瘤标志物的特异性，包括其它疾病，特别是感染性疾病；②通过技术的进步，找到针对肺癌不同病理类型的更加特异的肿瘤标志物；③联合几种不同的肿瘤标志物进行诊断。
